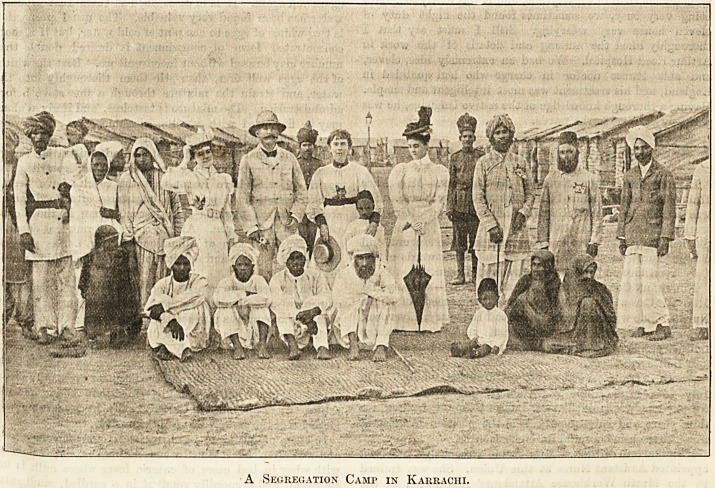# "The Hospital" Nursing Mirror

**Published:** 1899-08-05

**Authors:** 


"The Hospital" Nursing Mirror.

Being the Nursing Section of "The Hospital."

[Contributions for this Section of "The Hospital" should be addressed to the Editor, The Hospital, 28 & 29, Southampton Street, Strand, London, W.C., and should have the word "Nursing" plainly written in left-hand top corner of the envelope.]

### Notes on News from the Nursing World.

### THE ROYAL RED CROSS.

The coveted honour of the Royal Red Cross has been bestowed by her Majesty upon Miss Leonora Maxwell-Müller, lady superintendent of the Indian Nursing Service in the Madras Presidency, and upon Miss Annie Gill Mark, superintendent Army Nursing Service, and Miss Gertrude Mary Payne, Army Nursing Service. In the case of the two latter ladies the honour is in recognition of their services in tending the sick and wounded. In that of Miss Maxwell-Müller it has been given in recognition of her competency in the discharge of her duties, and in the care bestowed in training British soldiers and Army Hospital Corps attendants in nursing duties. Miss Maxwell-Müller was trained in the General Hospital, Birmingham, and after holding various appointments in the Royal Scotch Nursing Institute and in the Guards Hospital, London, she entered the Indian Nursing Service in 1889. She became deputy-superintendent, and in 1893 was subsequently appointed lady superintendent of the Madras presidency. Miss Maxwell-Müller was at one time a student at the Ear Hospital, Glasgow, and holds the certificate of the London Obstetrical Society. She is also a registered member of the Royal British Nurses' Association.

### CHILDREN AT THE ROYAL ISLE OF WIGHT INFIRMARY.

The people of Ryde and of the island must be congratulated on the new wing of the Royal Isle of Wight Infirmary, which was opened by the Queen last Friday. In her kindly and sympathetic speech on the memorable occasion the Sovereign expressed the great satisfaction with which she performed the ceremony, and her gratification that special provision had been made to extend to children the benefits which the older patients have enjoyed. We are quite sure that the nurses, as well as the patients, at the infirmary, will extremely appreciate the erection of a ward for the reception of children only. The most ardent lover of the little ones who ever put on the uniform of a nurse must recognise the value of the separation of children from grown-up folks wherever it can be effected. They know that it is good for both that they should not be in the same wards; that the conditions of nursing are different; that the relatives of children want special watching; and that in many other ways the latter can be treated much better when they are by themselves, while the gain to weak and weary men and women exhausted by long illness and slowly struggling back to health is too obvious to need insisting upon. Some day, it may be hoped, there will be no hospital or infirmary of any size without a ward set apart entirely for children.

### LADY HOWARD DE WALDEN.

By the death of the Dowager Lady Howard de Walden, the Nurses' Co-operation, 8, New Cavendish Street, has lost a sterling and liberal friend. She manifested a practical interest in the new nurses' home now in process of erection for the members of the society. Having first given the site and a considerable sum towards the building fund, her own architect being charged with drawing the plans, she herself exercised a keen supervision in the progress of the work. She was looking forward to being present at the opening, which will take place in the course of next year. Every member of this society regarded her with affection and esteem. Lady Howard was in her ninety-fourth year. She inherited three large fortunes on the death of her brother, the late Duke of Portland, and of her sisters, Lady Harriett Bentinck in 1882, and Lady Ossington in 1889.

### NURSING REFORM ABROAD.

The reopening of the Royal Military Hospital, Athens, under the direction of English Sisters, has already been announced. Owing to the great drought only part of the hospital is yet in working order, the wards not in use being closed until the autumn, when the welcome rain is expected. A short sketch of how things were done before the advent of the English Sisters may (says a correspondent) be of interest to those who think of taking up nursing abroad. It may also supply an idea of how much there is to be undone. "Under the old *régime*," she continues, "the head orderly reigned supreme, going round with the doctors, and receiving all orders as to treatment, &c. Rules and regulations were unknown, all directions being carried out in the most haphazard fashion possible. Old wine and beer bottles did service for medicine bottles. These stood on the lockers, the patient taking a 'pull' at the bottle when thirsty. Those too ill to help themselves fared worse, for the orderly would seize the bottle, and say, 'Open your mouth,' and toss a dose down the unfortunate man's throat! The night orderly's first notion was to select a comfortable bed in the ward, and much aggrieved he felt if his rest was disturbed by the calls of a patient. In the morning medicines and powders were reported as given; in reality they had been consigned to the lavatory. Much indignation has been expressed at the new order of things. The first lot of orderlies working under supervision asked to resign *en masse*; and on being questioned as to their reasons they replied: 'In all our lives we have never been used to anything like this—rules, order, punctuality, and cleanliness. Why, we would rather be in prison—no smoking in the wards, and not even allowed to keep our bread under our mattresses!' Anyone visiting the hospital as it now is would find it difficult to believe that things were once as related. Orderlies have ceased to rule the wards, black beer bottles have given way to proper medicine bottles, and the thirsty patient can no longer refresh himself *ad lib.*, for all medicine bottles are locked in a neat glass case in the middle of the ward. A good deal still remains to be done; but who can despair after the thin edge of the wedge has been inserted?"

### "ARISTOCRATIC HOSPITAL NURSES."

UNDER this heading an article appears in a contemporary, and "the head surgeon of a big London hospital" is credited with the assertion that there "is quite a remarkable impulse just now among young women of culture and education to become hospital nurses," and he more definitely affirms that "the Countess of Aislie's daughter, Lady Griselda, some time ago joined an Edinburgh hospital as probationer; while only lately he has received quite thirty applications from ladies, some being clergymen's and doctors' daughters, two Girton ex-students, and several others highly connected." But who is "the Countess of Aislie"? The Countess of Airlie cannot be meant. As for the rest of the statement, members of the aristocracy are always welcomed warmly at the hospital. There is no more truly, and, in the best sense, democratic institution than a big public hospital; and culture and education, with everything that increases knowledge and refinement, are most important possessions to a nurse, though the daughter of obscure parents is welcomed as warmly by the matrons of such institutions as the daughter of parents who can trace their ancestors to the Conquest.

### WESTMINSTER HOSPITAL.

SOME of the wards of the Westminster Hospital are closed for cleaning, and also to enable a few structural alterations to be made. These for the most part are intended to supply the nurses with improved sleeping accommodation, which is rather badly needed. The staff in the hospital numbers nearly sixty, and much care and foresight is required to provide for the physical welfare of so many. The matron, taking advantage of the slackened pressure on the work, is giving her nurses their holidays. The trying weather of the past few weeks has made it very desirable that all who can should get away from town. For this reason, and also because the Westminster Hospital Nursing Home has a private pension fund of its own, comparatively few of the staff were present at the brilliant ceremony at Marlborough House last week. The home to which nurses are drafted who are suitable for private work and who wish to continue in connexion with the hospital which was their training school, offers a pension of £20 per annum to each nurse who has rendered twenty years' service. Applications for admission as probationers to the hospital are, we learn, very numerous.

### NURSES AND BICYCLES.

A SUBSCRIBER to the Banbridge Nursing Society is very indignant that some of the members should have proposed to purchase a bicycle for the use of the trained nurse. She states that the nurses preceding the present one all purchased their own "bikes," and she sees no reason why an exception should be made. But circumstances alter cases, and if the present nurse has not a bicycle, cannot afford to purchase or hire one, and yet finds it a necessity in fulfilling her engagements expeditiously, it seems ungracious to protest against her being provided with one. There is, too, clear room for compromise. Why should not the Banbridge Society buy the bicycle for themselves and lend it to the nurse? Then it would do for her successors. This view of the matter may not have occurred to the members of the organisation. If human suffering can be alleviated more promptly, and possibly human lives saved, in the north of Ireland or elsewhere, by the use of a bicycle, the question of precedent should not stand in the way.

### A HEROIC NURSE.

THE New York papers give full accounts of an act of heroism on the part of Nurse Norah D. Abbe. She was in charge of the babies' ward of Bellevue Hospital one night last month, and in her efforts to save her charges she burnt three fingers on the right hand and scorched one on the left. Before assistance had arrived this brave woman had extinguished the blaze on all the beds save one. The netting and clothing of eight beds were destroyed, but the tiny patients in the ward are none the worse for their experience. Nurse Abbe has been at Bellevue Hospital since December, 1896, and has therefore still six months before she completes her training. Her parents were among the pioneers of Elyria, Illinois, the prairie State, and she is but 22 years of age.

### FEMALE HOSPITALS AND NURSES IN INDIA.

FROM the Rajputana Sanitary, Vaccination, Dispensary, and Jail report for 1897 we glean some interesting details about the work that is going on in female hospitals in India. It is a pity that the report is not issued before the summer of 1899, but Anglo-Indian officials do not hurry themselves. The climate is against it. At the Lady Dufferin Hospital, Ulwar, we notice that 11,787 patients were treated and over 2,504 operations performed. At the Jaswant Hospital at Jodhpore a Calcutta qualified female hospital nurse was appointed early in the year to assist Miss Adams, M.D., but the report states that she would not remain at Jodhpore although a good salary and well-furnished quarters were provided for her. A qualified nurse was therefore appointed to reside in the hospital, and she gives valuable assistance. Two girls from Ulwar qualified as maternity nurses during the year, and *dhais* have, it appears, been instructed by some of the leading doctors to enable them to work scientifically among the people. But the report significantly adds, "However, as most of them are quite illiterate, their instruction is no easy task."

### FETE ON BEHALF OF THE SOUTHGATE NURSE FUND.

IT is getting on for four years since Southgate first had a district nurse for the sick and poor. At the outset she was looked upon as quite an innovation. People shook their heads, and said it would not last long. However, the funds increased year by year, fresh subscribers came forward, and the residents showed their interest by sending old linen and parcels of warm clothing. On Thursday last week the Southgate Nurse Fund attained its red-letter day. Through the kindness of Mr. and Mrs. C. H. Fieling a grand fête was held in the grounds of Southgate House. Rich and poor worked hard to make it a success. The tickets went like wildfire, and Queen's weather favoured the great day. Mrs. Frank Bevan, of Trent Park, in opening the fête, testified in encouraging words to the keen interest she takes in the fund. The President (the Rev. J. Beardall) in his speech, emphasised the advantages of being a thoroughly well-trained nurse, and drew attention to the good work the nurse (Miss Thomas) is doing. When expenses were paid the sum of £105 was realised, and all felt that unity and interest in the good cause had more than fulfilled expectations.

### THE ARMY AND INDIAN NURSING SERVICES.

The *Morning Post* of Delhi says that a proposal is on foot to amalgamate the British and Indian Army Nursing eSrvices. It says that a period of training at Netley and some experience in military nursing in home hospitals would greatly add to the efficiency of ladies coming out to nurse the sick soldier in India. This opinion is widely shared at home.

### THE CATHEDRAL NURSE AND LOAN SOCIETY.

There is an erroneous impression in some parts of the North of England about this excellent organisation which Miss Coleman, the active head of it, has done her best to remove. The title of the society is simply due to the fact that it was started by Canon Lloyd, now Bishop of Thetford, to minister to the poor in their own homes in the parish of St. Nicholas, Newcastle-on-Tyne. But there is not, of course, any kind of religious test. As Miss Coleman puts it, there are only two qualifications the patients need. They must be poor, and they must be sick. Starting with one nurse in 1882, the association now has eight in full work. As an instance of the gratitude of some of the patients, a poor woman begged one of the nurses to accept half-a-crown for the society, which she had "saved up" for the purpose.

### CURIOSITY AND INFECTION.

The spectacle witnessed the other evening of a number of persons at their doors in a narrow suburban street, including several children, watching for a patient to be carried into an ambulance, serves as a reminder of the dangers of curiosity. Some of the onlookers, more venturesome than the rest, had crept up as close to the van as if it had been a carriage waiting to take a bride to church, and it did not appear to occur to the driver that it was at least a little risky, especially for children, to gather round his vehicle. The case was one of scarlet fever. It is possible, of course, that when the nurse descended she recognised the danger and urged the people to go away; but prevention is better than cure, and it would be a wise precaution on the part of a nurse in charge of a hospital ambulance to warn the driver not to allow inquisitive bystanders to come near to it.

### MEN AS MISCHIEF MAKERS.

It is generally supposed that women are more apt to make mischief than men. This supposition is not confirmed by the proceedings at a special meeting of the Mossley Sick Nursing Association the other day. It is not necessary to discuss|the details, which have apparently caused a certain amount of irritation among the members of the organisation. But Mr. Wright Mosley, in commenting upon the situation, affirmed that "the association was very well managed when it was left solely to the ladies. They began to fall out only when the gentlemen began to interfere." Holding this opinion, Mr. Mosley naturally urged that the association could be best managed in the hands of the ladies entirely; and if he is correct as to his facts, in this particular instance the sexes might be better divided. But nursing associations, as a rule, are not managed by one sex, and the ladies usually rather welcome than resent the suggestions of men.

### NO ENTRANCE FEE FOR PROBATIONERS AT BELFAST.

Mrs. Fennell, a member of the Belfast Board of Guardians, proposed at a recent meeting that all probationers should be charged an entrance fee of £5 a year, and the proposal was seconded by Dr. M'Donnell. It was urged that as the result of a similar proposition being carried into effect in another institution in the city the managers had obtained a better class of nurse. But the guardians did not take kindly to the idea, and it was rejected. One of them insisted that they ought not to put a heavy barrier in the way of a young woman who could become of good service to the community by making it binding upon her to pay an entrance fee. There is force in this, though Mrs Fennell rejoined that if a girl had all the other qualifications it would not be difficult for her to raise £5. Mr. Allison, however, made a very curious speech. He said: "Surely they had not to learn again the lesson of the Crimea, when the aristocracy pensioned off their sons by purchasing commissions for them. The principle of purchase had passed away, and the principle of efficiency had come, and had come to remain." But it was not suggested that the entrance fee should be a substitute for efficiency.

### LAURELS FOR THE ACCRINGTON NURSES.

In a competition which took place the other day in South-East Lancashire for a furnished box of "first aid" appliances the Accrington nurses, though the smallest division in the field, defeated all their rivals for the second time. The Oldham nurses were second, and those of Bury third. Naturally, the winners are very proud of their laurels, which reflect not only much credit upon themselves, but also upon the superintendent of the Accrington Nursing Association.

### THE ST. NEOTS AND DISTRICT NURSING ASSOCIATION.

At the annual meeting of this association the Chairman (Dr. Meade) said that it had proved of the greatest value to the district. He himself had enjoyed ample opportunities of proving the good work which the nurse was doing among the poor, and he found that they were loud in praise of the nurse and grateful to the association for the help afforded them. Moreover, they demonstrated their gratitude by sending small subscriptions to its funds. A desire was expressed that Eynesbury should join the association, and hearty thanks were accorded to the general committee for starting and managing it so successfully.

### SHORT ITEMS.

On Saturday Lady George Hamilton opened the new wing of the Victoria Hospital at Deal, which was enlarged in commemoration of Her Majesty's Diamond Jubilee; and subsequently she presented the St. John Ambulance certificates.—The treasurer of Guy's Hospital has received a further donation of £5,000 to the re-endowment fund from "M."—St. Mary's Hospital has had a bed endowed. The donor is Mr. L. E. Raphael, whose cheque for £1,000 was sent to the secretary for that purpose.—Miss Erskine, late of the Darwen Fever Hospital and District Nursing Institution, was last week unanimously appointed superintendent of the Grimsby and District Nursing Institution.

### Gynæcological Nursing.

By G. A. Hawkins-Ambler, F.R.C.S., Surgeon to the Samaritan Free Hospital for Women; Assistant Surgeon to the Stanley Hospital, Liverpool.

(*Continued from page 232.*)

### AMENITIES OF THE SICK ROOM.

The amenities of the sick room consist of little offices which, though trivial in themselves, add up into something considerable for the comfort of the patient and the relief of suffering. The well-intentioned nurse may or may not understand how best to serve the patient by doing the right thing at the right time. When it is no longer the right thing, what would otherwise tend to the relief of pain and weariness becomes a source of irritation and disquietude. She will, in the first place, study her patient. Some people choose best to be left alone, and unless they are to be disturbed for what is absolutely essential they prefer not to be tended at all. To such as these the nurse sitting rigidly by the bedside, or keeping her attention closely fixed on a patient, gives one the idea of a gaoler in charge of a criminal rather than the gentle servitor of the afflicted. It must, like so many other things, be left to the tact and the intuition of the nurse; but, though she does not have these qualities very strongly marked, natural intelligence and sympathy will teach her all she requires to know. A patient who is wearied, for example, and sleepless, may often be refreshed or encouraged to fall into restoring sleep if her hair be gently brushed or her face and hands sponged with tepid water. If the room be hot a little unobtrusive use of the fan will be of service. The administration of foods and medicine may be carried out without unnecessarily exerting the patient. An occasional word of comfort or reassurance will encourage her to wait with such patience as she can command for a hopeful issue of her troubles.

After operations women often suffer a good deal from abdominal pain, which is oftener than not caused by flatulence, and this can often be considerably relieved by the application of a light rubber hot water bottle, which can be moved from place to place for the local application of soothing heat. Indeed, it is sometimes amusing to see a patient chase her pain all over the abdomen with one of these useful little accessories. Everything that is done for a patient should be done with a quiet regularity which shall not excite or weary her. I have known nurses, very ready to criticise the doctor in attendance, who invariably left a patient uncovered and steaming in a not over-warm room after removing a poultice and while making a new one.

Sickness after the administration of anæsthetics may often be considerably relieved by hanging a sponge wrung out of vinegar close to the patient, and the intense thirst, which is a symptom of shock amongst other things, is more easily borne if the patient have placed over her lips a towel that has been dipped in vinegar.

There are times when a patient is encouraged by being chatted with, and others when conversation is simply destructive of all rest and a source of infinite irritation. I have known nurses chatter the whole night through to patients who were simply dying for want of sleep, and not merely talking for the sake of talking, with the soothing monotony and regularity of a curtain lecture, but, by a diabolical ingenuity, cultivating a conversation which called for incessant replies from the suffering listener.

I never yet found a patient satisfied with her food. She always wants solids when liquids are the only things that are safe for her, and she will probably grumble at anything that you bring her; but you can make an unpalatable diet less repulsive by serving it daintily, and you may sometimes induce a patient to take two small cups of beef tea when a small basinful would have made her refuse it altogether.

Such attention to the toilet of the patient as can be safely given helps a good deal to beguile the tedium of the day if it is judiciously spread out. This does not mean, of course, that you are to insist on every detail of the toilet if a patient is not in a condition to bear it.

Then the ventilation of a room requires considerable intelligence in its contrivance; and where a heated, close room would keep a patient hot and sleepless all night a chamber that has been cooled and thoroughly ventilated will frequently promote calm and refreshing sleep.

The orderliness of a room is also worth considering. Apart from the fact that disorder means waste of time and of temper, it is more restful and soothing to women, who are supposed to be the personification of domestic order, to observe that the usual accessories of the sick room are in their proper positions, and are kept clean and tidy.

While it is good for your patient to know that you are at hand for any service she may require, it is also good for her to feel that she, after all, belongs in some measure to herself, and is not living under the bondage of her attendant. Let her feel sometimes that she belongs to herself, and as convalescence progresses let her feel this more and more.

Some patients are naturally querulous and selfish, and cannot bear to see you with an unoccupied moment. With such as these, while you must not omit any necessary duty, you must be sufficiently firm, and not permit yourself to be worried by the thoughtless demands of the selfish and inconsiderate. It is not good for them, and it is certainly exhausting for you both. But an exhibition of unwillingness and surliness is not the way to make these patients more manageable. Being naturally of an irritable, nervous temperament, any impatience on your part excites and confuses them, makes them more and more unmanageable, and keeps their brains in a fretful condition, which has its effect on their physical state, and is reflected in numberless maddening demands on your time and temper.

### Suppositories.

Medicines that have to be administered by the bowel are usually made up in the form of suppositories, with which most nurses are familiar.

After greasing your finger, press the point of the suppository against the anus and push it gently well within the rectum. In cases where there is any affection of the lower bowel which prevents the retention of the medicament you would, after inserting, apply a pad of absorbent wool, and support it with a "T" bandage.

Nutrient enemata are frequently given where we desire to support the patient's strength without making any unnecessary demands on her digestive powers or interfering with the quietude of the upper bowel. The formulæ for some of these enemata will be given later on. It is just as well here to say that before administration it is a wise plan to give an injection of plain warm water; this cleanses the bowel and makes it more certain to absorb the enema, and also removes from it unabsorbed material, which is apt to irritate and make it less retentive of nutrient injections. If the water is merely absorbed it is of no consequence; it will probably be beneficial to the patient. After the prolonged use of these enemata the bowel sometimes becomes very irritable and will not retain them without the addition of a few drops of laudanum to each injection. This must not be administered without the permission of the surgeon. Sometimes it is necessary to inject a small enema of plain starch with laudanum to soothe this irritability. But the careful predigestion of the food to be so given and the preliminary washing out of the bowel will go far to promote the successful prolonged use of what is an invaluable method of sustaining strength.

### A Year's Plague Nursing in India.

By a Sister.

### DECLINE OF THE EPIDEMIC.—NURSING IN ARTHUR ROAD HOSPITAL.

The epidemic in Karrachi was a short but very severe one, and it began to subside in July. I noticed that the buboes seemed sometimes of rather a different character to those I had seen either in Poona or Bombay. They had more of a tendency to become gangrenous, which may have been often due to applications that the natives had used before being discovered and brought into the hospitals. These buboes were very troublesome and slow to heal, but there were some very successful cases. Before closing my account of Karrachi I must refer to the very great kindness the nurses received from the English people living there. Very soon after we arrived several ladies called on us, and although they could not of course enter the hospitals, which would have been neither wise nor safe, they insisted upon taking us out for lovely drives nearly every evening, which we thoroughly enjoyed. We were made members of the Ladies' Club, to which we could go any day, and I am sure we received invitations to dinners and "at homes" two or three times a week. Everything was done for our amusement and enjoyment when we were off duty, and we thoroughly liked both our nursing and our social life there. Indeed, we were very sorry when the fiat went forth that our duties were at an end and that we were to return to Bombay, which was our headquarters. At the same time it was a great pleasure when we were leaving Karrachi to see only about thirty patients in our wards, instead of every shed and room full to overflowing of plague cases which we had had in all stages of severe illness.

About this time plague had decreased considerably all over India. The heat was intense, and it was always in the hottest months that we noticed a great change, while as the days began to get colder the number of plague cases increased. This is no doubt owing to the natives sleeping out of doors during the hot weather, and therefore breathing purer air. We had a dreadful sea journey from Karrachi to Bombay during the monsoon weather, and one which I shall never forget. We experienced all the horrors of sea-sickness during the two days and nights we were travelling. Although the heat in Karrachi is trying, yet there is always a sea breeze which makes it bearable, but it is a climate which is wonderfully conducive to "prickly heat," from which we all suffered cruelly when we were there. On returning to Bombay we again experienced that nasty, hot, sticky, moist heat which is so disagreeable and irritating, and although it is fairly pleasant after sunset, owing also to the sea breeze, still the clammy feeling is always in the air. Fortunately, we enjoyed a long holiday during the most unpleasant time of the year, and on arriving at the hotel to which we had been ordered to proceed we found twenty nurses already there off duty, and also enjoying a rest and holiday, for which we were all thankful. Living at a large hotel would have been very expensive for us, but according to the rules of our agreement we were always provided with furnished quarters and we had to board ourselves; but as special arrangements had been made for us at the hotel, and there were so many of us living there, the proprietor reduced the charges for the nurses, and our expenses were no greater than if we had been living elsewhere. We soon, however, got tired of doing nothing. Hotel life is rather monotonous, and owing to the extreme heat we were unable to go out all day, and consequently time hung rather heavily on our hands. I was not sorry when, with some other nurses, I was ordered on duty again. This time I was sent to Arthur Road Hos-

A Segregation Camp in Karrachi.

pital, which is well known to most of our English nurses, and which formerly was one of the hardest worked plague hospitals in India. Certainly it was, in my judgment, the best organised. The building was made of stone with a roof of tiles supported on an iron framework which projected beyond the walls. This allowed of plenty of ventilation, and a good space was also open between the walls and floor, the latter being made of stone and built deck fashion, which could easily be washed and quickly dried. Not having many plague cases at this time, there were only three nurses working at this hospital, which, by-the-bye, was one of many in Bombay, and was kept principally for Hindoos and Mahommedans. The Mahrattas, who were of a different caste, had a hospital specially for themselves. Our hours on and off duty were much the same, except that our turn for night duty came once every fortnight for one week at a time. This we found very trying, especially as the mosquitoes and other small animals abound then, and were a great deal more troublesome than in the daytime, and it was lonely too. Not being very busy, we sometimes found the night duty of eleven hours very wearying. Still I must say that I thoroughly liked the nursing and details of the work in Arthur Road Hospital. We had an extremely nice, clever, and able Parsee doctor in charge who had qualified in England, and his treatment was most intelligent and simple. Having a thorough knowledge of the native language, he was perfectly at home in the wards.

### Minor Appointments.

LLANELLY DISTRICT NURSING ASSOCIATION.—Miss Alice Hulme has been appointed Assistant Maternity Nurse. She was trained at Chester General Infirmary, and afterwards worked on the private staff for one year and four months. She has since been private nursing on her own account, and charge nurse of the women's and children's wards of the Carnarvonshire and Anglesey Infirmary, Bangor.

NORTHERN CONVALESCENT FEVER HOSPITAL.—On July 21st Miss H. Carswell was appointed Superintendent of Night Nurses. She was trained at Mill Road Infirmary, Liverpool, and has since been superintendent nurse at Alcester Infirmary, Warwickshire, and superintendent nurse at Rochford Infirmary, Essex.

HOSPITAL FOR WOMEN AND CHILDREN, LEEDS.—On July 16th Miss Annie A. Symonds was appointed Staff Nurse. She was trained at St. Luke's Hospital, Halifax, for three years, and subsequently held the position of assistant nurse at the same hospital for eight months.

HUNGERFORD UNION.—Miss Annie Greenhalgh has been appointed Assistant Nurse at this Union. She was trained by the Meath Workhouse Attendants' Association at the Crumpsall Infirmary, Manchester.

ISLINGTON INFIRMARY.—Miss Louisa Canner has been appointed Assistant Nurse. She was trained by the Meath Workhouse Attendants' Association at the Royal Bucks Hospital, Aylesbury.

LEOMINSTER COTTAGE HOSPITAL.—On July 26th Miss Ellen Harrington was appointed Nurse Matron. She was trained at University College Hospital, London, and subsequently held an appointment in the same institution.

BRADFORD UNION WORKHOUSE HOSPITAL.—On July 26th Miss Elizabeth Martin was appointed Charge Night Nurse. She was trained at Kensington Infirmary, and subsequently held the post of charge nurse at Bradford Union Hospital.

COUNTY HOSPITAL, RYDE.—On July 21st Miss Newman was appointed Charge Nurse. She was trained at the Nottingham General Hospital, and has since done private nursing at Bexhill-on-Sea.

CRAWLEY AND IFIELD COTTAGE HOSPITAL.—Mrs. Fittall has been appointed Nurse Matron. She has held appointments at the Cottage Hospital, Caterham, &c.

### On the Use of Egg Albumen in Illness.

BY SISTER ELIZABETH.

PERHAPS it may interest some of the readers of THE HOSPITAL engaged in the practical nursing and supervision of the sick to hear a little about the free use of raw white of egg in the diets of certain medical cases in a ward of thirty women and children in a large general hospital. In such a ward a very large proportion of the more chronic cases are youngish women suffering from anæmia, gastric ulcer, and dyspeptic troubles of a more or less severe character, with their attendant miseries. Even with the greatest care in feeding, and the most gradual approach to solid food, the relapsing nature of these chronic complaints is but too apt to assert itself, and the wearisome round of nutrient enemata, hourly ounces of peptonised milk, and so forth has to be resumed again and again. It is in cases such as these that albumen water has been found very valuable. The usual proportion is two whites of eggs to one pint of cold water, but if a more concentrated form of nourishment is desired double that number may be used without inconvenience. Beat the whites of the eggs well first, then stir them thoroughly into the water, and strain the mixture through a fine sieve before administration. The mixture is tasteless, and if given alone may be flavoured with vanilla, cinnamon, &c., but when given in milk and whey better unflavoured.

From personal observation of the administration of egg water to patients suffering from dyspepsia, gastritis, and gastric ulcer, I have learnt that the results have been a quicker cessation of pain and uneasiness after food, and a steadier march towards convalescence than in those cases where it was not given. After a course of nutrient enemata a teaspoonful or two of albuminous water, *i.e.*, egg water, every hour is a safe and nutritious way of beginning mouth feeding again.

In three cases who were having large enemata of ten ounces of peptonised milk every six hours, the addition of the raw white of an egg was made with good results; there was an entire absence of diarrhœa and discomfort—a great gain, as all these cases were fed only by enemata for ten days or a fortnight. In a fourth case the addition of white of egg made no special difference, and the enemata were only moderately retained; but it should be added that the patient was taking two teaspoonfuls of Carlsbad salts every morning, so a looseness of the bowels was to be expected.

In cases of obstinate vomiting, egg water is very useful, and will often be retained when nothing else is; combined with whey in bad cases of enteric fever where milk is not tolerated, and is speedily vomited in a curdled, undigested condition, it forms a good food for some days, till milk can be resumed. Taken in its concentrated form (four whites of eggs to the pint) it proved of the greatest service to a young woman suffering from a severe attack of enteric fever in the above-mentioned ward, all sickness stopping after its administration, and the strength being well maintained.

Children with diarrhœa and vomiting have benefited by taking it alone and in conjunction with whey, when it has been advisable to stop milk for a time. Stimulants may very well be diluted with albumen-water instead of plain water, in cases where it is desirable to increase the nutrition.

Egg-water should not be added to boiling or even to very hot liquids, as the rapid coagulation of the albumen under heat will at once render it indigestible, and negative the hoped-for good results.

It is well known in France as "Eau albuminense," and one is inclined to surmise it to be a "good remedy out of fashion," though none the less valuable on that account.

The experiences of others who may have used egg-water as an article of diet for the sick would be of great interest to the nursing world, and especially to the writer.

### Echoes from the Outside World.

### AN OPEN LETTER TO A HOSPITAL NURSE.

Last week there was much talk of the probabilities of an outbreak of hostilities in South Africa, but I am told by some of my men friends that the firm and unmistakable speeches of Mr. Balfour, Mr. Chamberlain, and last, but not least, the Prime Minister, have done a good deal to make for peace. You know that if, unhappily, President Kruger should still persist in refusing the Uitlanders the rights which everyone here, except those funny subjects of the Queen who pride themselves on being Little Englanders, admits must be conceded to them, there will be war, and war would mean as much excitement and anxiety to nurses as to any class, partly because of the immediate demand by the Colonial Nursing Service for more help, and also because many of you have friends, or relatives, in South Africa who could not fail to be affected by a struggle between the British and the Boers. Of course, we Englishwomen are perfectly satisfied that victory would sooner or later be with the British, but, even so, what a fearful lot of suffering there would be on our side as well as on that of the enemy. We can only all hope and pray that such a terrible catastrophe may not occur, and that Oom Paul, who is supposed to be a very religious man, and is certainly a very sanctimonious one, will not violate the first principles of religion by provoking a conflict between races who should live in peace with each other.

---

The London Season has come to an end. I feel bound to tell you this, in my capacity of Chatterer-in-Chief, but it can make very little difference to most of you. The hospitals are just as full, however empty fashionable London may be. Sickness does not stop its march across the land because lords and ladies have gone to foreign watering-places to get cured of ills frequently produced by late hours and over-excitement; and to most of you the principal effect which August has is to cause you to long more than before for your own holiday, if it is yet to come, and to wish it were not over if it is, alas! already in the background. There is one little matter which always causes me a pang of regret whenever I think about it, or it is brought prominently before me by meeting a man out of livery, with a straw hat on his head, driving a lovely pair of horses, his roomy carriage either unoccupied or only filled by his wife and children or his fellow-servants. Why, oh, why do not some of the kind-hearted women who make up our aristocracy—and there are lots of them who would be only too pleased to do a kind deed if someone would remind them that the deed were waiting to be done—leave orders that, say, three times a week the carriage is to go for orders to the matron of one of our big hospitals, in order that nurses, worn out with the heat, might get a little restful refreshment, or that patients who are far advanced towards convalescence might be further strengthened and benefited? Had I a carriage, or two or three, as the owners of many establishments have, it would give me so much pleasure to be able to share my good things, as it were; but then, perhaps, if I belonged to what the poor people call "kerridge folk" I should, like them, forget.

---

Do you see that the Rev. George Richardson has prophesied that in fifty years' time the great public schools would be mixed schools for boys and girls? His opinion is worth having, for he has been second master at Winchester for thirty-three years, and on Tuesday, when he was presented with some plate and jewellery by the Old Wykehamists, he confessed that he had become a convert to the mixed system. Of course, when he began his tutorial career girls who were able to keep pace with clever boys in scholastic attainments were greatly in the minority, because the higher education of women was only just being mooted. But during the many years he has watched the progress of events he has seen the girls creep up and take the coveted positions, till now they are not only B.A.'s and M.A.'s, but M.D.'s and senior wranglers. Personally, I can see nothing but good to come from the mixed system at colleges. Necessarily, the boarding-houses for girls and boys would be distinct, and they would meet principally in class only. But the mere fact of working so much together would do more than anything else, I believe, to get rid of silly giggling flirtations of girls and boys, and encourage instead the spirit of friendship and rivalry which is so much to be desired.

---

Decidedly, margarine seems getting into bad repute. It is a delightfully elastic name, and I suppose, as long as the magic piece of paper announcing that the compound is not sold as pure butter is forthcoming, there is nothing to prevent the margarine being as impure as the manufacturers care to make it. From evidence which I came across the other day it appears that poor people do not patronise it nearly as much as big institutions and firms who cater for a great number of employés. Shop assistants are frequently treated to bread and margarine, and one convent in Northamptonshire takes about 60 lb. a week. Whether the devotées and the children whom they educate flourish upon the fare is not known, but the statistics of one of the asylums where the patients have an ounce a day given to them are not in favour of margarine. In this particular asylum the deaths are 30 per cent. in excess of the rate in three neighbouring asylums where butter is substituted for margarine. In another instance, for thirteen months butter only was given to the asylum patients; then margarine was tried for nine months. Immediately the death-rate rose considerably. Of course, such statements are not conclusive arguments, but at least the evidence against margarine looks suspicious. That the composition can be made with little, if any, disagreeable taste is proved by a recent case at Bristol, where a woman used to buy 200 lb. of margarine per month, work it up into round pats to resemble butter, print an acorn on the top of each, and, in the garb of a countrywoman, with a basket and clean white cloth, successfully sell the pats to many customers as "fresh country butter." Her profits—which must have been considerable—will be a good deal reduced, if not quite swallowed up, by the £20 fine, and costs, which she has been ordered to pay.

---

An amusing incident has happened at Chicago. The child of fashionable parents had the whooping-cough, and consequently she was obliged to forego the many amusements to which she had been accustomed. But her enforced quietude gave her time to think, and the result was that, after making inquiries amongst all the numerous children of her acquaintance, she discovered that twenty-seven other young people were suffering from the same malady. Thereupon she promptly asked them all to come to a whooping-cough party. It is said that they enjoyed themselves vastly, but it is a little difficult to accept this as a fact. If active games were played all would cough at once; if quiet ones the "barking" of two or three patients would quite prevent the others listening, and, as fits of whooping-cough are often attended with inconvenient results, it is not easy to look upon the gathering as a festive one. I am glad that at present our English children take their whooping-cough more soberly, and do not turn it into the occasion for holding a novel entertainment.

### A Sister's Post from Two Points of View.

VIEW I: THE PRO'S.

As on Mount Pisgah's lofty height
I viewed the post of "Sister,"
I thought of it with awe as great
As works of Treves or Lister.
For I was yet so new a pro.
That glad anticipation
Still coloured all my new career
Of lofty aspiration.

Ah, me! I thought, if only I
Could rise to that position,
I think I should entirely reach
The height of my ambition.
No beds to make, no "Brights" to clean,
No lockers to be scrubbing,
No crying babies to console,
No patients to be tubbing.

No one to say I make mistakes,
No Sister by to scold me,
And ask me why I did not do
Exactly as she told me.
No one to "mark" me when I'm late
For breakfast or for dinner,
And make me feel there never yet
Existed such a sinner!

If *I* could have a sitting-room
How charming I could make it;
If only I could get the chance
How gladly would I take it.
I then should have my lunch at one,
And dine at half-past seven;
And even have my friends to tea
Or coffee at eleven!

And very often then I'd get
A Saturday to Monday;
I'd go out visiting, or else
I'd stay in bed all Sunday.
Oh, how I wish the years were passed,
And I certificated,
That I might prove if things were just
What I anticipated.

If I were but a Sister I
Am sure I'd never worry,
I'd never be the least bit cross,
And never in a hurry.
I'd always treat my nurses too
With great consideration,
Remembering from day to day
The time of my probation.

I'd be so good to all my pro's
They'd simply idolise me,
And even carping critics should
Forbear to criticise me.
I'd be—oh! dear, there's Sister's voice,
"This baby's bottle's leaking;
Nurse, come and wipe up all this mess,
Quick! don't you hear me speaking?"

VIEW II.: THE SISTER'S.

Ah, well! the years are past and gone,
I've got the post I prayed for;
The post I was so sure that I
Especially was made for.
But full enough I find it now
Of every sort of trouble;
I thought I'd much in bygone days,
But now I've fully double!

The easy time I thought I d have
Has proved a big delusion,
My grand ideas have vanished in
A kind of wild confusion.
I used at least to give account
Of only *my* misdoings,
And had not nurses then to train
And ponder all their goings.

I'm now the scapegoat of the ward,
With sins of each one on me;
No matter who has done the wrong,
It's visited upon me.
Has something been forgotten? Why
Did Sister not see to it?
Or something left undone by nurse?
Why didn't Sister do it?

The patients and the patients' friends
All wish to interview me,
And each one thinks his own affairs
The most important to me.
The keeping of the books alone
Drives me well-nigh distracted;
I'd no idea from anyone
So much could be exacted.

If I should plan an afternoon
To have some recreation,
Quite often they will fix that day
To do an operation.
If everything is not just so
They don't forget to blame me;
But when I happen to be right
They do not even name me!

Whatever other folks may do
The Sister must be ready
For every fresh emergency,
With quiet nerves and steady.
If fifty interruptions come,
She still must keep on smiling;
And be the ideal comforter,
The weary hours beguiling.

To keep up discipline all round
She always must be able;
And still unruffled calm preserve
Amid a very Babel.
No matter how the children cry,
And rend the air with squalling;
No matter what may tumble down
And break with noise appalling.

Her cupboards must be always neat
And ready for inspection;
Her dressings must be up-to-date,
Her nurses all perfection.

House surgeons even may forget
Sometimes, but Sister never!
If *she* forgets she hears of it
For ever and for ever.

She must remember every fad
Of surgeon, clerk, or dresser,
Supply for each the thing he likes,
The greater and the lesser.
Treat every ward-clerk with respect,
Nor smile at any error;
He *may* get "on the staff" some day
And make himself a terror!

The dressers may be very slow,
But Sister must not say so;
House surgeons may come very late,
She mayn't say, "Why delay so?"
Is any cross? She must use tact,
And seek the fault to cover,
Not answer back, nor notice it,
But try to gloss it over.

'Tis true she has her sitting-room,
But little time to use it,
And if an invitation comes
She often must refuse it.
The post *is* hard: why did I toil
So desperately to win it?
I thought I used to work, but now
I find I wasn't "in it"!

Whichever way I look at it,
It's not an unmixed blessing.
Exciting? No, at times it's dull,
I do not mind confessing.
And if I ever let myself
Become less energetic
I quickly find my nurses too
Are getting apathetic.

And yet about the work there is
A certain fascination,
Which makes one feel, it's, after all,
An ideal occupation.
Perhaps it's having every day
Filled full to overflowing
Which makes us all so wide awake,
And keeps our interest growing.

Perhaps it is the little ones
In helplessness appealing
That makes us long to bring to them
The needed gift of healing.
When wasted limbs grow firm and round
And little faces brighten
We feel we cannot do enough
The children's lot to lighten.

When sick and dying, young or old,
For help and comfort sighing,
Stretch out to us their trembling hands,
On our support relying,
'Tis then we know wherein it lies,
This power of fascination;
In helping those who need our help
We find our compensation.

E. M. Fox.

### The Opening of a New Wing of the Royal Isle of Wight Infirmary.

IMPROVED ACCOMMODATION FOR NURSES.

To the great delight of the Ryde people the Queen opened the new wing of this institution on Friday. The town was gaily decorated for the occasion, and special trains and excursion boats brought thousands of visitors. The new building, which is connected with the old hospital by a covered way, consists of a spacious entrance corridor, lined with cupboards and lockers for the children's clothes, bath room, ward kitchen, charge nurses' room, and the ward, with rooms overhead for the necessary increase to the nursing staff. The ward is well finished in every detail, the floors are teak, the corners are rounded, and the ward has a sun room at one end also floored with teak, while the sides are entirely composed of glass so that the children will get the benefit of light and sunshine even during the cold winds which keep them indoors. The nurses' rooms are four in number and are furnished with pretty suites in birch, the floors being stained. The new rooms are furnished in exactly the same way as the rooms now in use in a wing which was built for the nurses' accommodation in 1895. Only two are double-bedded, and nearly all the nurses, now fifteen in number, have, therefore, separate bed-rooms, The cost of the wing has been about £3,300, and the money has been contributed by inhabitants of all classes, and from all parts of the Isle of Wight. It has provision for ten cots, and five have already been endowed at a cost of £1,000 each.

Prior to the arrival of the Queen on Friday evening, Princess Henry of Battenberg, president of the hospital, unveiled a life-bust of the Queen by Mr. Onslow Ford, R.A. Her Majesty was received with Royal salute, the band playing the National Anthem. The fire brigade also lined the ground, and a large platform within the grounds accommodated about a thousand invited guests. The Queen remained in the carriage, which drew up alongside a raised daïs where Princess Henry of Battenberg stood awaiting the arrival of her mother. The Deputy-Governor, who was presented by the Princess, stepped forward and read an address in which it was set forth tnat the new wing of the hospital had been erected by the inhabitants of the island as a memorial of their loyal devotion to Her Majesty's throne and person, and of their thankful sense of the blessings which the country had enjoyed in Her Majesty's reign. After briefly recording the good effected by the institution during the past and preceding years, the address went on :—

"Your Majesty's gracious presence here to-day confers an additional honour on the hospital, which already owes so much to your Majesty's generous support and encouragement throughout its career of fifty years of ever-increasing usefulness; and this occasion will be regarded as yet another sign of the continued interest which your Majesty is now graciously pleased to show in all that concerns the welfare of the inhabitants of the Isle of Wight."

The Queen read the following reply :—

"I thank you for your loyal address. It gives me great satisfaction to open the children's wing of the Royal Isle of Wight Infirmary and County Hospital. I have never ceased to watch with interest the growing usefulness of this institution, and I am gratified that special provision is now being made to extend to children the benefits which older patients have enjoyed. I am glad to associate this event with the completion of the sixtieth year of my reign, and I receive with pleasure your renewed expression of attachment to my Throne and person."

A hymn, "Thou to whom the sick and dying," having been sung by the choir of the parish church, Princess Henry of Battenberg made several presentations to Her Majesty. The Bishop of Winchester offered a prayer, after which the Queen, amid cheers, opened the door of the new wing by touching a lever which completed an electric circuit. Three cheers were then raised for the Queen on the call of the Deputy-Governor, after which the Royal party returned to Osborne.

### Royal Infirmary, Preston.

A TRIP OF NURSES AND MEDICAL STAFF TO THE LAKES.

BY A CORRESPONDENT.

THIS most enjoyable event took place last month, Mr. Park, chairman of the board, being the kind and genial host. Starting in the special saloon provided on the 6.15 a.m. train, they commenced their journey to Windermere. On arriving at Lakeside everyone leaving the train embarked on one of the lake steamers and got what was, to many of the party, a first glimpse of the English lakes. Arriving at Bowness, breakfast was attacked with an appetite little short of phenomenal, and everyone remarked that the house surgeon accompanying the party showed distinct signs of recovery from the attack of sleepiness consequent on his early start. After breakfast the party again set sail, and in due course arrived at Ambleside. A visit was paid to Stock Ghyll Force, which was particularly grand after the rains. The amateur photographer now showed signs of renewed activity, and insisted upon taking a group on the rocks below the falls. After almost super-human efforts the group was formed, the camera focussed, best smiles donned, and the shutter raised; what the result will be still remains to be seen. Upon returning to Ambleside a coach was found ready to take us to Grasmere. The drive through Wordsworth's own particular country was very lovely, and was enjoyed almost more than any other event of the day; the poet's seat and cottage, duly pointed out by the driver, came in for a proper amount of observation. Luncheon over, the party separated into groups, some visiting Wordsworth's tomb in Grasmere Churchyard; while others, developing a sudden ardour for mountaineering feats, started off for the nearest hill. How far up they got we did not inquire; they looked too hot and tired to be questioned. On getting back to Ambleside the route on the other side of Rydal Water was taken, and this seemed almost more picturesque than that bv which we had come. After a row on the lake the return to Bowness commenced. Tea was here partaken of, and a vote of thanks to Mr. Park being proposed by the deputy-chairman, and seconded by one of the staff, our host briefly replied. We next proceeded to drag our tired legs up to Windermere Station, but on arriving half-an-hour too soon the more energetic members of the party rushed up to the top of Orrest Head, where a last glimpse was caught of the beautiful lake we were so regretfully leaving. The home journey being successfully accomplished everyone arrived, tired certainly, but only sorry to think that one of the most delightful days we had ever spent was over. Too much praise cannot be given to Mr. Park for this outing; during the whole day he was indefatigable in his efforts to ensure the utmost enjoyment of all. Would that there were more chairmen like him.

### Presentations.

MOGDEN HOSPITAL, ISLEWORTH.—Miss Annie Carter, on the occasion of her leaving, was on July 24th the recipient of a charming pair of pictures from the nursing staff. The domestic staff presented Miss Carter with a handsome travelling clock.

CARNARVONSHIRE AND ANGLESEY INFIRMARY.—Nurse Alice Hulme, charge nurse of the women's wards at this infirmary, was presented upon leaving with a handsome leather chatelaine by the probationer nurses, who most sincerely wish her every success in her future work.

### Everybody's Opinion.

[Correspondence on all subjects is invited, but we cannot in any way be responsible for the opinions expressed by our correspondents. No communication can be entertained if the name and address of the correspondent is not given, as a guarantee of good faith but not necessarily for publication, or unless one side of the paper only is written on.]

### THE PARK FEVER HOSPITAL.

"ONCE A PRO," who sends a letter respecting this hospital, omits to enclose her name and address. Until she complies with this rule her communication cannot be published.

### NURSES' READING SOCIETY.

"MISS MOBERLEY" writes: I hear from one or two members that they have been unable to obtain the book for this quarter, "The Treatment of Phthisis," by Dr. Ransome, as it is just out of print. Perhaps nurses who have not obtained it will wait to join the society until next quarter, October 1st, and I will date their subscriptions from then if they will communicate with me. One nurse asks me what the 2s. 6d. subscription is used for? May I point out that a large number of examination questions will have to be posted quarterly, also corrected papers, and further, that the management of a society like this involves a considerable amount of time and labour which the moderate subscription cannot be said to pay for.

### A RESTFUL PLACE FOR A HOLIDAY.

"A NURSE" writes: Now that the holidays have set in it would perhaps please many of your readers to know of a quiet spot which is yet near many places of interest. I refer to Brinklow, a village about eight miles from Rugby, where I spent a fortnight last September. The Lodge, a quarter of a mile from the station, is a home from home, with an old-fashioned garden in which one can only be lazy. Coventry is eight miles distant, with its legendary town and famous church, the longest in England. Rugby has its school and Arnold Chapel: Lutterworth, all its Wycliff associations and the wonderful fresco in the church of the resurrection dating from the twelfth century. Warwick and Kenilworth are so full of historical interest that they are worthy of a whole day's visit; while Stratford-on-Avon is an easy distance to any ordinary cyclist. Indeed, I think one could not have a more interesting holiday, and the lanes of Warwickshire are very refreshing to tired workers. It can also be worked with very little expense, which means everything to a nurse.

### NURSING IN POOR LAW INSTITUTIONS.

MISS LOUISA TWINING writes: It must be a matter for regret to all who have the welfare of the sick poor at heart, to find that this subject was not included in the discussions on "Nursing," in the recent International Conference of Women. Surely no department of the question can exceed in importance that of how to obtain the benefits of real nursing for the thousands of helpless and afflicted persons who are now the chief occupants of our poor law infirmaries, and that it was not even named appears a remarkable omission. However, all that remains to be done is now to continue to bring forward the facts and conditions of the existing state of thing, and to reiterate them on every possible occasion and opportunity. Amongst the most valuable of these evidences is that of the excellent nurses who have long been employed in the service of the poor law, and are acquainted with a state of things wholly unknown to outsiders, and even in a great degree to the guardians who visit the wards, and are supposed to be familiar with the arrangements. I am much surprised if a further knowledge thus obtained will not confirm the conviction, long entertained by many, that at least one efficient remedy for the chaos and ignorance that now prevails, will be the increased appointments of women inspectors, to whom alone the real conditions of nursing and nursing grievances can be made known, and thus, it is to be hoped, remedied. As nothing can help us in our efforts for reform like plain and truthful statements of facts, I venture to ask you to give publicity to the following extracts from nurses now working in poor law infirmaries, and in whose competence and characters we have the fullest confidence and trust. But I cannot help repeating the oft-told conviction that no remedies can suffice while the control of highly-trained, and often highly-educated, women is given to untrained men and women, the present officers of these institutions. I cannot help asking, would any other profession or trade consent to be placed under unskilled and untrained foremen or forewomen, who yet are called their "superiors"? Under such conditions it is inevitable that friction must exist, even with the most patient and long-suffering, frequent changes in the staff being, as guardians well know, the certain result. Separation, where possible, of the infirmary, under the control of the head nurse, seems the only possible remedy, unless the more drastic one of amalgamation of the smaller workhouses into classified institutions be adopted. But, after all, surely the first and great need is for the Central Board to take up the question seriously, to make it a special department, with women as advisers, and adopt some plan and standard of training, to fix a period of probation, and after that a term of service, so that those trained in infirmaries should not, as at present, frequently transfer their services elsewhere, but, as in the army and navy, be bound for a certain number of years, with a pension to be granted at the end. The present well-nigh impossibility of obtaining a sufficient number of probationers will never be overcome till some such system is adopted. For the ever-increasing demand for nurses at home and abroad, with far superior advantages and attractions, must reduce the number of applicants for posts so inferior in every way as those in poor law service. Funds must, of course, be provided, but salaries would not be expected by probationers in view of such advantages, while their services would benefit the institutions; and as many Boards expend large sums yearly on advertising, mostly in vain, why should they not contribute to a plan which would give them certain and thoroughly efficient help in their difficulties? The following is from a nurse who has done excellent previous work in reforming a country infirmary: "The sick here are so scattered, and one is bound to go out of doors to get to any of the wards. I have 52 beds, without the lying-in and receiving wards, which make another eight. I have only a night nurse in the same room by day that I sleep in at night. In the winter we do not know where to put the people; a new infirmary has been talked of for years, but nothing is done. The work is much too hard; but it would not be so bad if the patients were not so scattered—and I am obliged to depend on pauper help. If the night nurse were a trained nurse, so that I could talk to her about the work, it would be pleasanter; but she does not understand or care. I have no one I can speak to except when the doctor comes, which is twice a week. It seems as though I am working quite alone, and I never feel satisfied, for I know I do not do what ought to be done, because it is impossible to do it." Another nurse writes of the change of master and matron: "They have never had such a post before, and hence the cause of all our disappointments. Owing to their want of previous training and experience they lack the necessary qualifications to fill the post they hold. The diet from the first I complained of as quite unfit for the sick. My patients were served with the fattest, coarsest cut of mutton chops; I had taken back dinners and asked that the beef tea might be free from fat, and that we might have better and lean meat, but my request was not granted. The clothing is also very defective, especially for the old women, who feel the cold. . . . I cannot be still and see the old style of things going on and never make an effort to have things as I think they ought to be." I cannot help thinking, if all these matters were reported to the Central Board, as they might be by women inspectors, that some measures must be taken to amend them, and I ask again, who is so fit as a woman to make all these investigations, connected as they must necessarily be with the nurse's work? A well-known authority on this subject writes: "When the Local Government Board takes on itself to organise the great body of nurses which it employs, the perplexing problems which distract the profession will then come within measurable distance of being solved." That the Irish Board is moving in this direction may perhaps encourage ours to do likewise and take the lead in much-needed reforms. May I add that we should rejoice if more power could be given to the Central Board to enforce them.

### For Reading to the Sick.

FORTITUDE.

**Verses.**

NOR deem who to that bliss aspire
Must win their way through blood and fire!
The writhings of a wounded heart
Are fiercer than a foeman's dart.
Oft in life's stillest shade reclining,
In desolation unrepining,
Without a hope on earth to find
A mirror in an answering mind;
Meek souls there are who little deem
Their daily strife an angel's theme. —*Keble.*

Father, if we may well endure
The ill that with our lives begins,
May'st Thou, to whom all things are pure,
Endure our follies and our sins!

Brothers, if we return you good
For evil thought or malice done,
Doubt not that in our hearts a blood
As hot as in your own may run. —*Houghton.*

If call'd, like Abraham's child, to climb
The hill of sacrifice,
Some angel may be there in time
Deliverance shall arise!

Or, if some darker lot be good,
Oh, teach us to endure
The sorrow, pain, or solitude
That makes the spirit pure! —*Irons.*

Not light and momentary labour these,
But discipline and self-denial long,
And purpose staunch and perseverance asked,
And energy that inspiration seemed.
—*Pollok.*

God spake and gave us the Word to keep,
Bade never fold the hands nor sleep
'Mid a faithless world; at watch and ward
Till Christ at the end relieve our guard.
By His servant Moses the watch was set;
Though near upon cockcrow, we keep it yet.
—*Browning.*

**Reading.**

The gift of fortitude or strength, of courage, of love of endurance, is necessary to perfect the will. For if the will be soft, shrinking, inconstant and cowardly, it can never hold out under suffering. The words of the Apostle, "Labour like a good soldier," may be also translated "Endure hardness," that is, bear pain, rise up against difficulty, accept crosses, suffering, privation, hardship, whatsoever may come in the way of duty. Fortitude, therefore, is of two kinds; there is an active fortitude and a passive fortitude. The gift of fortitude is what we commonly call courage. We see it to perfection in the soldier saints of the Old Testament, in Josue, in Gideon, in David, in the Maccabees. They were what the world calls heroes, what the Church calls saints. . . . The perfection of fortitude is in its passive character. It is to be seen in the life and passion of our Blessed Lord. The Son of God, who never lifted His hand but in benediction, nor stretched it out but to be nailed to the Cross, is the perfect pattern of fortitude.

### Appointments.

WOLVERHAMPTON AND STAFFORDSHIRE GENERAL HOSPITAL.—Miss Minnie Leng has been appointed Matron. She was trained at Guy's Hospital, which she entered as lady pupil in 1894. She has since been night sister for six months, holiday sister in several wards, sister of Mary Ward, sister of Astley Cooper Ward, and assistant matron for eighteen months, doing the matron's work in her holidays and during three months' sick leave.

BRIGHTON WORKHOUSE INFIRMARY.—The appointment of Miss Isabel H. Myles is as Superintendent Nurse, not staff nurse. She held the post of sister of a male surgical ward at Birmingham Infirmary for two years, and home sister for two years.

### Notes and Queries.

**The contents of the Editor's Letter-box have now reached such unwieldy proportions that it has become necessary to establish a hard and fast rule regarding Answers to Correspondents. In future, all questions requiring replies will continue to be answered in this column without any fee. If an answer is required by letter, a fee of half-a-crown must be enclosed with the note containing the enquiry. We are always pleased to help our numerous correspondents to the fullest extent, and we can trust them to sympathise in the overwhelming amount of writing which makes the new rules a necessity.**

**Every communication must be accompanied by the writer's name and address, otherwise it will receive no attention.**

*Superintendent's Duties.*

(156) I have recently been appointed superintendent nurse in a workhouse hospital, and at the time of the appointment the duties were not stated. Will you kindly inform me what are the duties and privileges of a superintendent nurse where the matron of the union is not a nurse? Should the nurses and probationers under my charge, also I myself, ask off duty from either the master or matron, according to the rule obtaining before a superintendent nurse was appointed? I am anxious to perform my duties in a proper manner, and to act rightly towards the matron, nurses, and myself.—*Superintendent Nurse.*

The duties of a superintendent nurse are defined by the committee appointed by the Guardians for that purpose, and vary in each establishment. If you are uncertain about any point ask your committee for instructions, explaining your position in order that they may be able to frame rules in accordance with the requirements of the case.

*Nurses' Clubs.*

(157) I shall be so much indebted if you will tell me whether there are nurses' clubs or hotels in Manchester and Edinburgh. I do so dislike living in an ordinary hotel, as I spend a night or two in towns travelling north or coming south again. If there are please give me the addresses. —*Clubs.*

Perhaps some of our readers may be able to advise "Clubs" in this matter.

*Diabetic Cookery.*

(158) Would you kindly give address of the publishers of G. Abbott and Son's "Cookery Receipts for Diabetics"?—*J. W.*

Messrs. George Van Abbott and Son, 6, Duke Street Mansions, Grosvenor Square.

*Old "Hospitals."*

(159) Will you kindly advise what best to do with five years' editions of THE HOSPITAL, unbound? I shall be grateful for any suggestion. Am willing to give them away if carriage were paid.—*M. L.*

The old copies of THE HOSPITAL and "Mirror" are so valuable for reference that you could not do better than have them bound. Apply to the Manager if you wish to do this.

*Housekeeper.*

(160) Can you inform me how to obtain a post as housekeeper or matron of institution in the Colonies? I am an experienced housekeeper, but not trained in nursing.—*Flight.*

The Secretary, the Emigrants' Information Office, 31, Broadway, Westminster, S.W., will give you reliable information. You should consider to what colony you wish to go, and also select the climate most suitable to your constitution before taking definite steps.

*Maternity Work.*

(161) Can you kindly tell me if there is any chance for a certificated maternity nurse, recently trained at the British Lying-in Hospital, not L.O.S., and with no general training, obtaining work at a fair salary. A parish, institution, or garrison town, either in or out of London, where there would be plenty of practice, would not be objected to. There must be no expenses attached to the appointment.—*Energy.*

If "Energy" will glance through our advertisement columns she will see how essential some general training in nursing in addition to her monthly training is. If she be young enough she would be well advised to train thoroughly in a Poor Law institution. In the "Mirror" for July 22nd, for instance, the Guardians of Keighley advertise for a probationer at a salary of £5 for the first year, £15 for the second, and £20 for the third. This is very good, as there are no expenses incurred, and on the completion of the training many posts will be open to the nurse thus qualified.

*Knowledge.*

(162) I have received my certificate for "first aid to the injured" from St. John Ambulance Association, and I am now awaiting the result of our Home Nursing examination for the same, but being desirous of still further knowledge, will you please be kind enough to tell me what book I can get in which to study all the different complaints and diseases and their treatment. Being a new reader of THE HOSPITAL, I must say I enjoy reading it very much. It is a very interesting and instructive book.—*New Reader.*

We thank "New Reader" for her encomium. No one book could possibly contain information whereby all complaints and their treatment could be studied. But there are several good books on the general principles of nursing that she would find useful. The Manager, the Scientific Press, would send her a price list on application. We note that a new volume is about to be issued, entitled "Handbook for Nurses," by J. H. Watson, M.D., M.B., C.M. (price 5s.); whilst a new edition of "Nursing: Its Theory and Practice," by Lewis, an old favourite, is to be ready shortly; the price is 3s. 6d.

*Nurses' Hotel.*

(163) Kindly give address of nurses' hotel in London, also rules connected with it, and charges?—*Nurse C.*

The Nurses' Hostel, Francis Street, W.C. The rules are those of an ordinary hotel, and the terms from 17s. 6d. a week for board and cubicle. It is comfortable and convenient.

### Travel Notes.

By Sister Grace.

### XXX.—BARNARD CASTLE, DURHAM.

I very seldóm recommend English places as holiday resorts for nurses, for the simple reason that they are too expensive for slender purses. It is a shame that such should be the case, and perhaps some day hotel keepers and lodging-house mistresses will see how emphatically it is to their own interest to bring down their prices and stop the steady flow of holiday makers to the continent. Conveniently placed for those living north, it struck me as being a very nice place in which to spend a week or fortnight if your home is in the north or temporary work confines you to that part. From London the railway fare is expensive—return ticket, third-class, £2 1s. 5d. ; but from Liverpool, Manchester, many of the northern or midland towns, this heavy initial expense would be largely reduced.

### Accommodation.

I stayed last year at the Three Horseshoes inn, and found it most comfortable, clean, quiet, and in all ways agreeable, but not cheap; if you think of spending an inexpensive holiday your best plan will be to go into lodgings, which abound everywhere, and many of them offer fairly reasonable terms.

### The Town and Castle.

The town itself resembles many another north country market town. In the Galgate, which runs up at right angles from Marwood Chase and the Flatts Woods, are the lodging-houses, on the road to the station. This Galgate is supposed to be the old place of execution, or gallows gate. The church is of some architectural interest ; it was built by Bernard Baliol in the early part of the twelfth century, and there is a fine Norman door on the side towards the town. Close by is the curious octagonal Town Hall, a delightful subject for artists and photographers. The chief entrance now to the Castle is through the gardens of the King's Head Hotel, celebrated in "Nicholas Nickleby." Nicholas was advised by Noggs to call there on account of the excellence of the tap. The position of the Castle, looking down upon the Tees, is very lovely. I spent much time inside sketching, and the loveliness of the view looking from an oriel window down upon Marwood Chase is well worth going far to see. The fortifications were destroyed by Cromwell, but sufficient remains of the massive walls to show what a place of importance it must have been.

### The Woods and Marwood Chase.

The walks through the woods are endless and ever-changing. One good walk is to descend the slope in front of Galgate and turn to the left, going under Baliol's Tower. This will bring you to the ancient town bridge, which bears the letters "E. B." and the date 1569, and is therefore supposed to have been built in the reign of Elizabeth. Cross this bridge and turn to the right till you come to a sort of suspension bridge, cross again and turn to the left, you will continue the walk through the woods till you pass the railway viaduct, when you turn into rich meadows, and still mounting return to Barnard Castle by an upper path.

### High Force and Cauldron Snout.

These falls are magnificent, and really easily reached. You must take the train early in the day to Middleton-in-Teesdale. From there an omnibus runs to Langdon Beck, but if you have cycles it is more agreeable. Four miles from Middleton you come to the High Force Inn. Very nice people here ; I should have liked to stay a week very much. Leave your cycles there and enter the path to the falls immediately in front of you by a small wicket. It is only about a quarter of a mile from the inn. After viewing the magnificent volume of water from below, mount to the top by steps cut in the rock. Do not, however, be tempted out on to the masses of boulders; a gentleman lost his life there some years ago by being swept over the falls. The Cauldron Snout lies about another five miles and a half up the dale, and is still finer than High Force.

### Rokeby is only distant four miles from Barnard Castle, and is an easy and delightful walk. The hall is occupied, and not often shown to visitors ; moreover, it is not of particular interest, but the walk through the grounds is lovely. Readers of "Rokeby" will be surprised to note the absolute local fidelity of Scott's account. He was visiting a member of the Morrit family when he conceived the idea of the poem. At the Dairy bridge which spans the Greta you turn up to the right to see Morthem Tower, where are still shown blood stains on the stairs, but their origin seems lost in obscurity. All is uncertain and mysterious.

"The 'lated peasant shunned the dell,
For superstition wont to tell
Of many a ghastly sound and sight
Scaring its path at dead of night."

### Eglistone Abbey is close to Barnard Castle, at the junction of Thorsgill-beck with the Tees, in the loveliest spot imaginable. It is a mere ruin, but enough remains for us to judge of its fair proportions. There are some very interesting flat tombstones, one with the inscription quite legible : "J. Rokeby, Bastarde" ; then comes a cross, and on the further side—

"Jesu, for yi (Thy) passion ser (sair),
Have mercy of yi sinful her (heir).

That tomb probably holds the secret of a very tragic life and death.

### Deepdale.

Every kind of walk can be taken around Barnard Castle, and none are more beautiful than those in Deepdale. A very good idea of the dale is afforded by a walk past the volunteer rifle range. The scenery is wild, lovely, and enchanting. You can go as far as the Catcastle rocks. Near by is an iron railway bridge, and at a short distance beyond a waterfall ; there is a path across fields leading from the railway viaduct which will take you to the village of Lartington, and thus home. There are endless excursions to be made with very little expense or fatigue. Notably that to Raby Castle close to Staindrop, an easy bicycle run ; Kilmond Sear, some two and a half miles distant ; Bowes, where in the churchyard you will see the grave of the ill-fated "Edwin and Emma," and many more that I must not stop to mention to-day. Altogether, the scenery around is rightly considered to be as fine as anything of its kind in England.

### TRAVEL NOTES AND QUERIES.

Rules in Regard to Correspondence for this Section.—All questioners must use a pseudonym for publication, but the communication must also bear the writer's own name and address as well, which will be regarded as confidential. All such communications to be addressed "Travel Editor, 'Nursing Mirror,' 28, Southampton Street, Strand." No charge will be made for inserting and answering questions in the inquiry column, and all will be answered in rotation as space permits. If an answer by letter is required, a stamped and addressed envelope must be enclosed, together with 2s. 6d., which fee will be devoted to the objects of the "Hospital Convalescent Fund." Any inquiries reaching the office after Monday cannot be answered in "The Mirror" of the current week.

Juan-les-Pins (Cosmo).—It is a very small place, close to Antibes, and would be an excellent spot for artists, cheaper and more agreeable than Antibes. Some very good excursions to be made from it. As for climate, much the same as Cannes.

Belgium (Inquirer).—No, Belgian French is not good. No doubt the upper classes speak well, as they have adopted it as their language, but as one mixes but little with this class in touring I should not recommend you to study French there, or, indeed, anywhere but in France. 2. It is most difficult to effect an entrance into a French family for residence ; they sternly set their faces against our intrusion into their domestic life. There are a few French Protestant pastors who take boarders, but there are generally other English, which neutralizes the benefit.

Carabacel (Ignorant).—Carabacel is a suburb of Nice—some people consider it the most agreeable part of the town. It is very sheltered and warm, but some distance from the sea and the Promenade des Anglais. The Hotel Hollande offers accommodation from seven francs, but it would be high up, and probably a shady room. The Hotel d'Europe et d'Amerique also offers from eight francs.

## Figures and Tables

**Figure f1:**